# Control of Cleft Glutamate Concentration and Glutamate Spill-Out by Perisynaptic Glia: Uptake and Diffusion Barriers

**DOI:** 10.1371/journal.pone.0070791

**Published:** 2013-08-12

**Authors:** Jean-Pierre Kessler

**Affiliations:** Aix Marseille Université, CNRS, CRN2M UMR 7286, Marseille, France; INSERM U901, France

## Abstract

Most glutamatergic synapses in the mammalian central nervous system are covered by thin astroglial processes that exert a dual action on synaptically released glutamate: they form physical barriers that oppose diffusion and they carry specific transporters that remove glutamate from the extracellular space. The present study was undertaken to investigate the dual action of glia by means of computer simulation. A realistic synapse model based on electron microscope data and Monte Carlo algorithms were used for this purpose. Results show (1) that physical obstacles formed by glial processes delay glutamate exit from the cleft and (2) that this effect is efficiently counteracted by glutamate uptake. Thus, depending on transporter densities, the presence of perisynaptic glia may result in increased or decreased glutamate transient in the synaptic cleft. Changes in temporal profiles of cleft glutamate concentration induced by glia differentially impact the response of the various synaptic and perisynaptic receptor subtypes. In particular, GluN2B- and GluN2C-NMDA receptor responses are strongly modified while GluN2A-NMDA receptor responses are almost unaffected. Thus, variations in glial transporter expression may allow differential tuning of NMDA receptors according to their subunit composition. In addition, simulation data suggest that the sink effect generated by transporters accumulation in the vicinity of the release site is the main mechanism limiting glutamate spill-out. Physical obstacles formed by glial processes play a comparatively minor role.

## Introduction

Most glutamatergic synapses in the mammalian central nervous system (CNS) are covered by thin astroglial processes that form the so-called perisynaptic glia. Electron microscope studies indicate that the fine structure of perisynaptic glia is extremely variable [1]. Depending on the synapse, glial processes may either wrap the entire synaptic diameter or only a part of it, and extend to various degrees over the pre and post synaptic elements. Intriguingly, strong variations are observed not only between synapses in different CNS regions but also between different types of synapses in the same CNS region and even between synapses apparently belonging to the same population in the same region [2],[3]. The nucleus tractus solitarii (NTS) a brainstem nucleus receiving visceral sensory information, contains both single synapses and multisynaptic arrangements. In a recent study, we showed that the glial coverage of single NTS synapses was 68% of the synaptic diameter on average but could vary from none to a nearly complete one [4].

The functional consequences of these structural variants are difficult to appreciate. Perisynaptic glia may act as a physical barrier opposing diffusion. Ultrastructural data confirm this view by showing that the space available for glutamate diffusion is reduced at synapses contacted by glial processes [4]. Thus, extensive glial wrapping may help prevent glutamate spill-out and subsequent activation of distant receptors but it may also impair glutamate exit from the synaptic cleft [5].

Perisynaptic glia also plays a major role in transmitter inactivation. Since there is no glutamate degrading enzyme in the extracellular space, inactivation exclusively depends on uptake by specific transmembrane carriers. Astroglial membranes express large amounts of the glutamate transporters GLAST/EAAT1 and/or GLT1/EAAT2 and carry out the bulk of glutamate uptake [6]. Thus, perisynaptic glia may reduce the possibility of activation of extrasynaptic receptors and of receptors located in neighboring synapses not only by creating barriers to diffusion but also by inactivating glutamate escaping the cleft. Glial uptake may also modifies intra-cleft receptor responses by altering the spatio-temporal profiles of glutamate concentrations within the cleft. Many simulation studies suggest that glial uptake does not change synaptic receptor activation [7],[8],[9]. However, experiments performed using transporter antagonists show that blocking glutamate uptake may also modify receptor responses [10],[11]. The effects depend on the type of receptor being investigated. While AMPA receptors (AMPAR) remain unaffected unless desensitization is blocked, glutamate transporter antagonists increase and prolong NMDA receptors (NMDAR) responses. Nevertheless, it should be kept in mind that enhanced NMDAR responses after uptake blockade may result from increased spill-out and action on distant receptors rather than from the prolonged presence of glutamate in the cleft.

The present study was undertaken to investigate the dual action of perisynaptic glia using computer simulation. The advantages of numerical simulation are twofold. First, simulation provides information on the effects of glia as a physical barrier to diffusion, which are not analyzable by other methods. Second, it allows to investigate the consequences of uptake blockade on intra-cleft receptors without any possible interference with effects on distant receptors. Effects of glia walls and transporters were analyzed using a synapse model reproducing the main characteristics of NTS glutamatergic synapses and Monte-Carlo algorithms to simulate glutamate diffusion and uptake.

## Materials and Methods

Simulation was carried out using custom-made programs written in C++, compiled and run on a Intel Xeon-based workstation (HP Z400).

### Model

**Figure 1:**
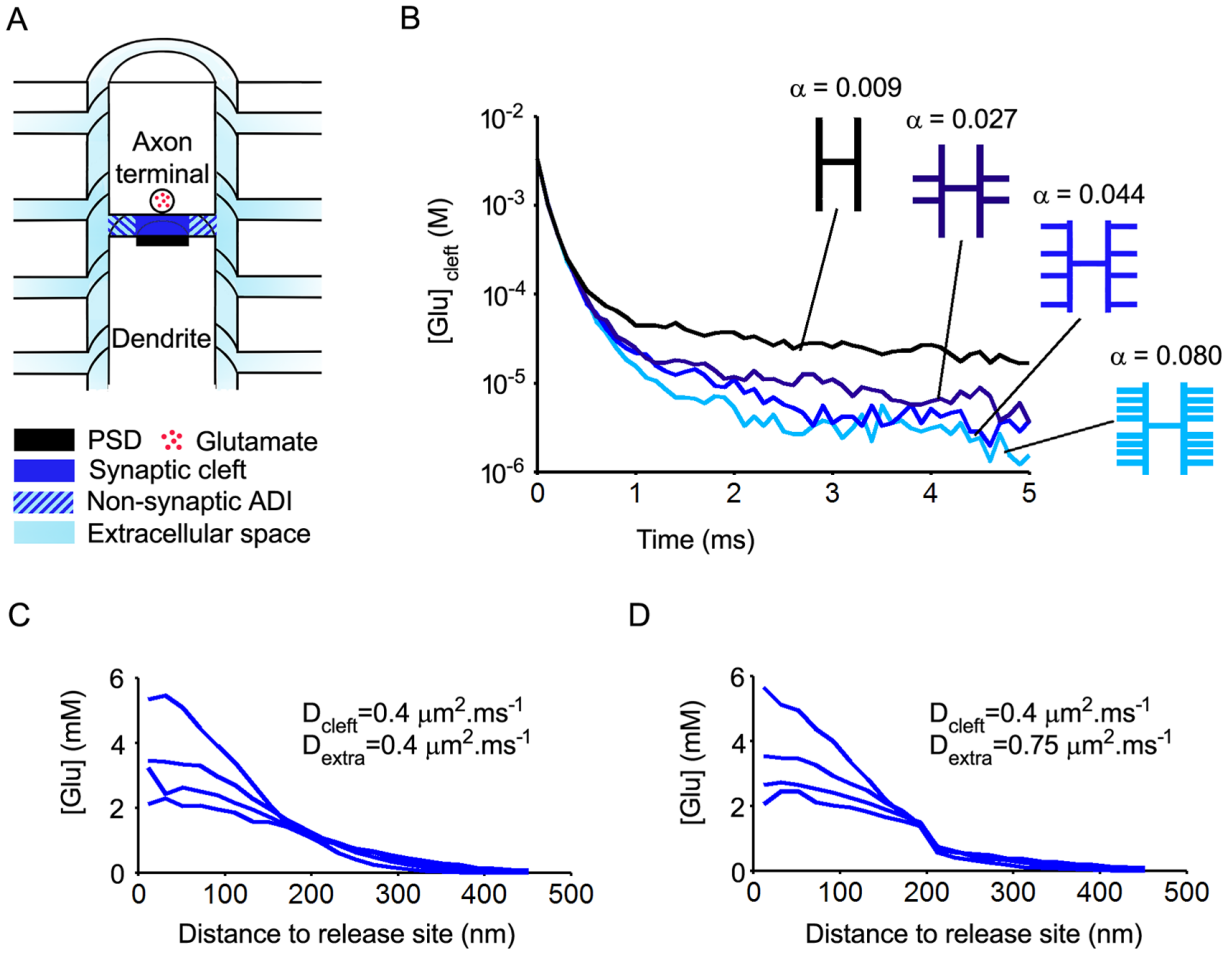
Effects of diffusion space geometry on the time course of glutamate in the synaptic cleft. A. Three-dimensional representation (half view, not to scale) of the synapse model. The diffusion space includes the axon-dendrite interface (ADI, radius: 500 nm ; width: 12 nm) divided into a synaptic cleft bordered by the PSD (radius: 200 nm) and a peripheral non-synaptic part, the extracellular space bordering the axon and the dendrite (width: 20 nm) and four escape routes orthogonal to the synapse axis (width of escape routes: 20 nm). Glutamate release occurs at the center of the ADI. B. Effects of model porosity. The porosity α is gradually increased by successive additions of escape routes. Each trace is an average of 5 trials. Glutamate exit from the cleft is strongly accelerated by the incorporation of the first two proximal escape routes. Further additions of escape route have less prominent effects. C and D. Effects of extrasynaptic diffusion coefficient (D_extra_) on spatio-temporal glutamate concentration profiles in the ADI. Traces were obtained at different time intervals after release (0.02 ms to 0.05 ms, each trace is the average of 5 trails). Increasing D_extra_ from 0.4 µm^2^.ms^−1^ to 0.75 µm^2^.ms^−1^ lowers glutamate concentrations in the non-synaptic part of the ADI but does not affect cleft glutamate content.

**Figure 2:**
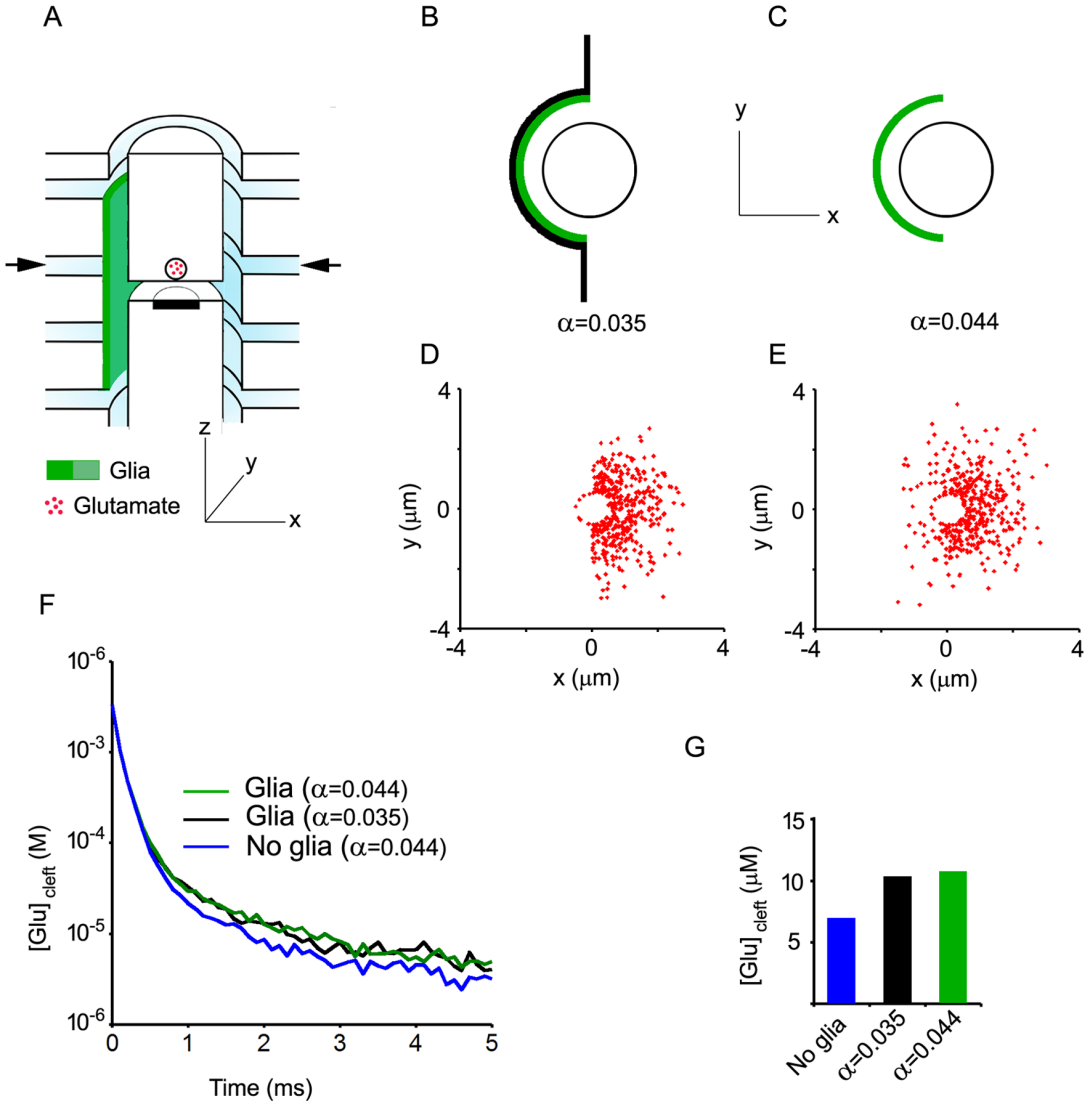
Effects of diffusion barriers. A, B and C depict the two conditions tested. In both conditions, proximal escape routes are partially obstructed by glial barriers (green). Arrows in A indicate the level of the top views shown in B and C. In B, diffusion barriers also extend within the escape routes thereby reducing the volume of the extracellular space available for diffusion (α = 0.035). In C, glial barriers are present around the axon and the dendrite (one half of the ADI perimeter covered) but do not extend within escape routes. Glutamate molecules must walk round the glial barriers to access the whole extracellular space but the actual porosity is not modified (α = 0.044, as in the no glia conditions). D and E. Snapshots of glutamate diffusion in the x-y plane indicated by arrows in A. They were obtained 1 ms after release using the glial barrier arrangements depicted in B and C, respectively. F. Time course of glutamate in the synaptic cleft in control conditions (no glia, blue) and with the glial barriers depicted in B (α = 0.044, green) and C (α = 0.035, black). Each trace is the average of 10 independent trials. G. Cleft glutamate concentrations averaged between 1 and 5 ms after release (means of 10 trials) with or without glial barriers. The two glial arrangements tested induce similar increases in cleft glutamate concentrations suggesting that they are functionally equivalent, at least within this time window.

**Figure 3:**
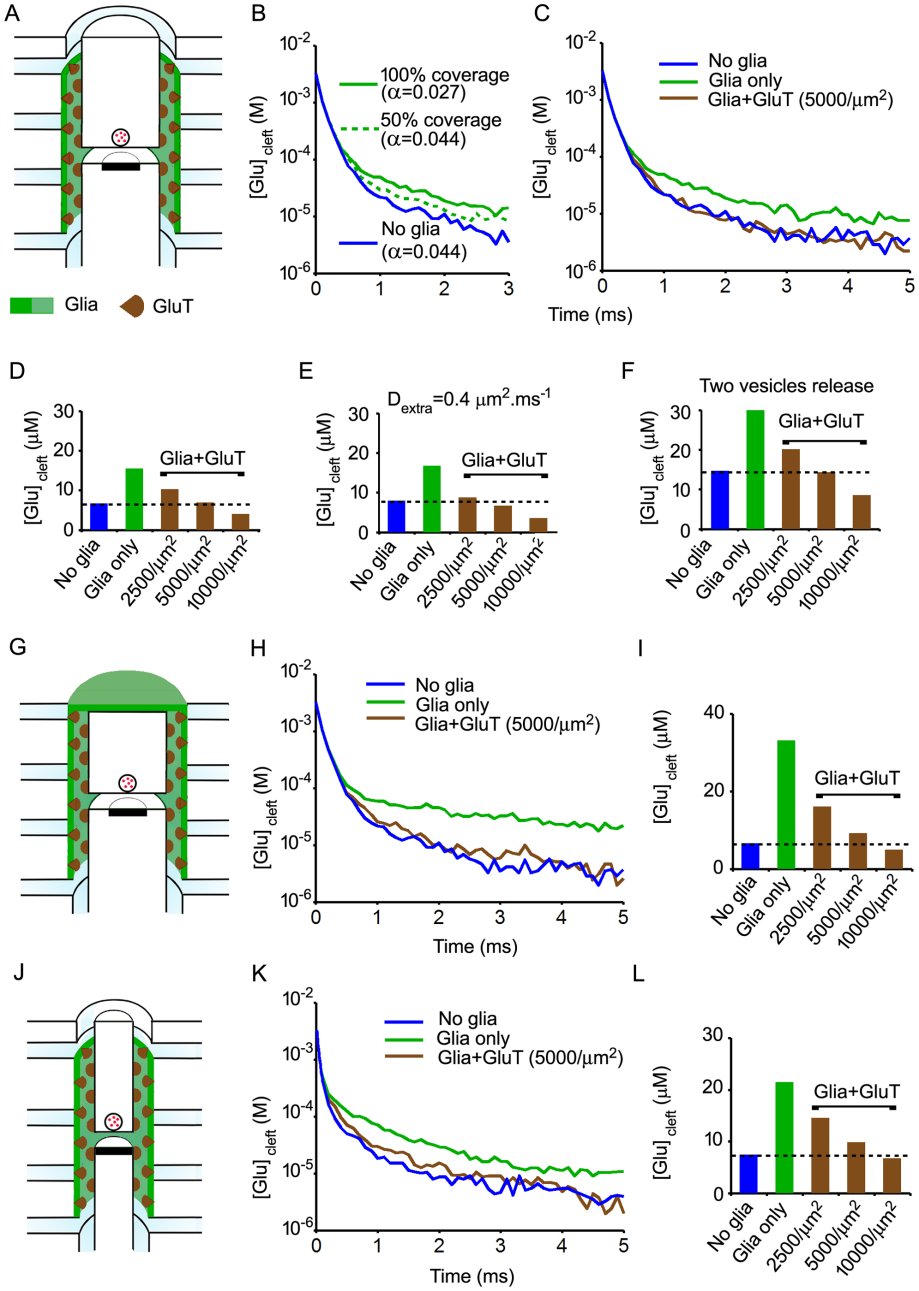
Effects of diffusion barriers and uptake on the time course of glutamate in the synaptic cleft. A. Three-dimensional representation of the model showing the localisation of glia and transporters (half view, not to scale). B. Time course of cleft glutamate concentrations without glia (blue curve) and with glial sheets surrounding either half of the ADI perimeter (50% coverage, green dashed curve) or the full ADI perimeter (100% coverage, green solid curve). C. Time course of cleft glutamate concentrations without glia (blue), with glial barriers (full ADI coverage) but without glutamate transporters (glia only, green ) and with both glial barriers and glutamate transporters (“Glia+ GluT”, brown). Each trace is the mean of 5 independent trials. D. Cleft glutamate concentrations averaged between 1 and 5 ms after release (means of 5 trials) with or without glia (full ADI coverage) and/or transporters. E. Same as in D but with coefficients of diffusion in the cleft and the extra-synaptic extracellular space set to the same value (0.4 µm^2^.ms^−1^). F. Same as in D but with simultaneous release of two vesicles (6000 molecules of glutamate released). G. Model with complete glial capping (half view, not to scale). H. Time course of cleft glutamate concentrations obtained using the model shown in G I. Cleft glutamate concentrations averaged between 1 and 5 ms after release (means of 5 trials). J. Model without non-synaptic ADI (half view, not to scale). K. Time course of cleft glutamate concentrations obtained using the model shown in J. L. Cleft glutamate concentrations averaged between 1 and 5 ms after release (means of 5 trials). Whatever the characteristics of the synapse, the amount of glutamate released and the disposition of glia, uptake by realistic glutamate transporters densities (5000/µm^2^ to 10000/µm^2^) compensate for the effect of diffusion barriers.

The synapse model (Figure 1A) was mostly based on quantitative information obtained by three-dimensional reconstruction of glutamatergic synapses from the NTS [4]. The glutamate diffusion space was modeled as a finite disk representing the axon-dendrite interface (ADI) continuous with an infinite hollow cylinder representing the immediate extracellular space around the pre-and post-synaptic elements. The ADI was divided into a central part corresponding to the synaptic cleft, i.e. the interface between the active zone and the post-synaptic density (PSD), and a peripheral non-synaptic part (non-synaptic ADI). Glutamate release was placed at the center of the cleft and the vesicular content was 3000 Glu molecules. The cleft and ADI radii were set to 200 nm and 500 nm, respectively (mean values obtained by three dimensional reconstructions). Further analysis of the 35 NTS synapses reconstructed in Chounlamountry and Kessler [4] indicated the height of the synaptic cleft was 12±2 nm (mean ± SD ; unpublished data). Thus, the height of the disk representing the ADI was set to 12 nm. The width of the extracellular space around the pre- and the post-synaptic elements was set to 20 nm. Extracellular space available for glutamate diffusion was increased by adding up to 8 infinite escape routes (20 nm wide) aligned perpendicularly to the synapse axis (Figure 1A, B). The most distal escape routes were placed at one micrometer from the ADI border. Thus, calculated extracellular volume fraction (i.e. porosity) with the 8 escape routes was 0.08, close to values obtained by measurements performed on electron micrographs in the vicinity of NTS synapses (0.09 after correction for 25% shrinkage). Analysis of diffusion pathways performed on electron micrographs from NTS synapses shows that the distance between the ADI edge and the next bifurcation of the extracellular space is 220 nm on average [4]. The most proximal escape routes were therefore placed at 200 nm from the ADI border on each side. Data obtained from actual NTS synapses also show that the distance between the ADI edge and the next bifurcation is greater (up to 1 µm or more in most cases) if perisynaptic glia is present [4]. The consequence of this is that fewer channels are available for diffusion in the vicinity of the synapse. Thus, the presence of glia was simulated in the model by partial or complete closure of escape routes and/or parts of the hollow cylinder (Figure 2 and Figure 3).

### Glutamate diffusion

Glutamate diffusion was calculated using the equations for Brownian displacement in a three dimension space:


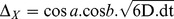
, 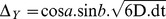 and 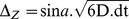 where dt is the elementary time step set to 10 ns, *a* and *b* are randomly generated angular values and D is the diffusion coefficient for glutamate. Two recent studies indicate that the apparent diffusion coefficient in the synaptic cleft is from 2 to 5 times lower than in free solution [12],[13]. Furthermore, electron microscope data show the presence of dense material between the active zone and the PSD, thereby suggesting that diffusion is slower in the cleft than in the non-synaptic extracellular space (see discussion). Therefore, the intra-cleft diffusion coefficient was set to 0.4 µm^2^.ms^−1^, i.e. approximately half the value for free diffusion. Outside the cleft, including the non-synaptic part of the ADI, the coefficient of diffusion was set to 0.75 µm^2^.ms^−1^ as in a free medium except otherwise indicated.

### Glutamate uptake

To simulate a glial location, glutamate transport sites were disposed at the external boundary of the hollow cylinder representing the immediate extracellular space around the pre- and post- synaptic elements (Figure 3). Except otherwise specified, glial wrapping surrounded the entire ADI perimeter. Glutamate transporter sites were homogeneously distributed in glial membranes. Glutamate uptake by glial transporter was calculated using a very simple kinetic scheme including a reversible binding step (k_on_  = 6.10^6^.M^−1^.s^−1^ and k_off_  = 500.s^−1^ ; [14]) and an irreversible trans-location step (k_trans_  = 500.s^−1^). Thus a bound glutamate molecule had the same probability to unbind as to be transported [14]. No relocation step was included in the scheme. Thus, each transporter site was able to take up one glutamate molecule only within each run.

To perform Monte Carlo calculation, the macroscopic binding rate *k_on_* was converted into probability of binding upon collision. This was done by dividing *k_on_*.*dt* – i.e. the number of binding events per time step and per M of glutamate – by the expected number of collisions per time step assuming a 1 M concentration of glutamate.

The number of collisions per time step (*N_col_*) is equal to half the number of glutamate molecules in the volume obtained by multiplying the transporter surface area by the mean glutamate molecule displacement per time step in one dimension. Assuming a 1 M glutamate concentration, *N_col_* is equal to: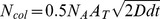 where *N_A_* is the Avogadro number, *A_T_* is the transporter surface area and *D* is the diffusion coefficient for glutamate in water. Thus, the probability of binding *P_on_* upon collision with a transporter is:



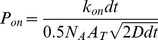



The transporter surface area *A_T_* was obtained by calculating the inverse of the transporter density (made variable between 1250 per µm^2^ and 40000 per µm^2^).

Macroscopic unbinding and trans-location rates were converted into probability using the following formula:


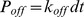
 and 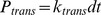


### Receptor activation

The ADI was divided in 20 nm width concentric rings centered on the release site. At each time step, the average glutamate concentration within each ring was calculated. No correction was made to account for receptor binding before calculating glutamate concentrations. Values obtained were used to determine the opening probabilities (*P_open_*) for AMPA and NMDAR and the activation ratio for mGluR1 (i.e. the ratio of receptors number in active states to total receptors number). AMPA receptor activation was calculated using kinetic schemes and rate constants from Robert and Howe [15] for GluA1- and GluA4-containing receptors and from Robert et al. [16] for GluA2-containing receptors. *P_open_* was obtained by calculating the ratio between the sum of open states concentration and the total receptors concentration using reducing coefficients of 1/3 and 2/3 for the di- and tri-liganded open states, respectively. NMDA receptor activation was calculated using kinetic schemes and rate constants from Erreger et al. [17] (scheme 4) for GluN2A- and GluN2B-containing receptors and from Dravid et al. [18] for GluN2C-containing receptors. *P_open_* was obtained by calculating the ratio between open states concentration and total receptors concentration. For GluN2C containing receptors, a reducing coefficient of 28/45 was applied to the first open state. Activation of mGluR1 was calculated using kinetic scheme and rate constants from Marcaggi et al. [19]. Typical synaptic and perisynaptic responses were obtained by averaging receptor *P_open_* obtained across the PSD and across the non-synaptic ADI respectively, assuming a homogeneous receptor distribution.

## Results

### Effects of model geometry on glutamate residence time in the synaptic cleft

Glutamate residence time in the cleft is in a large part determined by the extent and the disposition of space available for diffusion. Therefore, the first question addressed was to determine whether the model depicted in Figure 1A provides an accurate representation of glutamate diffusion in the actual micro-environment of NTS synapses. This was done by examining the effects of increasing space available for glutamate diffusion on the time course of glutamate in the synaptic cleft. Starting from a simple hollow cylinder connected to the ADI, increases in diffusion space were obtained by successive addition of escape routes (Figure 1B). Varying the number of escape routes did not alter cleft glutamate concentrations within the first 0.5 ms after release because diffusion within this time window is largely shaped by the properties of glutamate diffusion within the cleft environment [20]. Adding escape routes decreased cleft glutamate content past this delay. A strong decrease was obtained by incorporating two escape routes on each side of the ADI, close to its border. A weaker supplementary decrease was induced by adding two other escape routes distally (1000 nm from ADI border). Further incorporation of escape routes in between in order to increase extracellular volume fraction up to 0.08 decreased cleft glutamate content only slightly. Thus, in order to reduce computing time, the simpler model depicted in Figure 1A with 4 escape routes only was used for the subsequent steps of the study.

The effects of changing the speed of glutamate diffusion in the non-synaptic extracellular space were investigated next. This was done by examining the spatio-temporal profiles of glutamate concentration in the cleft and the non-synaptic ADI. It was found that decreasing the coefficient of diffusion outside the synaptic cleft from 0.75 µm^2^.ms^−1^ (as in a free medium) down to 0.4 µm^2^.ms^−1^ (cleft value) decreased glutamate transients in the non-synaptic part of the ADI but had little consequence on cleft glutamate content (Figure 1C,D).

### Effects of diffusion barriers

Electron microscope data indicate that the presence of glial processes around synapses results in less diffusion channels available for synaptically released glutamate [4]. Closing of diffusion channels by glia may be either complete, resulting in reduced extracellular volume fraction in the vicinity of the synapse, or only partial, leaving access to an unchanged extracellular space volume. These two possibilities were examined using the glial arrangements described in Figure 2A–C. In both cases glia sheets surrounded half of the synaptic perimeter obstructing parts of the proximal escape routes. As compared to the “No glia” conditions (α = 0.044), the volume of extracellular space available for diffusion was reduced in one configuration (α = 0.035 ; Figure 2B,D) but not the other (α = 0.044 ; Figure 2C,E). Insertion of glial barriers delayed glutamate exit from the cleft. The resulting increases in cleft content were noticeable 0.5 ms after release (Figure 2F). Unexpectedly, the increases induced by the two different glial arrangements were very similar both in time course and amplitude (Figure 2F,G). Thus, the two conditions, with and without an actual reduction of extracellular volume fraction, were equivalent as regards cleft glutamate content, at least during the time window investigated.

### Diffusion barriers versus uptake: effects on cleft glutamate content

The effects of combining diffusion barriers and uptake were examined next (Figure 3). Without uptake, increases in cleft content induced by the presence of perisynaptic glia depended upon the extent and disposition of glial membranes (Figure 3B). Up to sixfold increases were obtained with the extensive wrapping depicted in Figure 3G. Glial uptake efficiently counteracted the effects of diffusion barriers. Increases in cleft content were abolished (Figure 3C–F) or at least strongly reduced (extensive wrapping ; Figure 3H,I) by the addition of glutamate transporters at densities of 5000/µm^2^ in glial membranes. Furthermore, higher but still plausible transporter densities (10000/µm^2^) resulted in decreased cleft glutamate contents as compared to the “no-glia” conditions. Although absolute glutamate concentration values were two times higher, relative changes induced by glia and transporters were similar after single vesicle release (3000 molecules, Figure 3D) and after simultaneous release of two vesicles (6000 molecules, Figure 3F). Effects of glia on cleft glutamate content was also investigated using a smaller ADI (200 nm radius) devoid of any non-synaptic part (Figure 3J). The action of glia in this small synapse was noticeable 0.1 ms after release (Figure 3K). The net effect with transporter densities set to 5000/µm^2^ was a slight increase in cleft glutamate content (Figure 3L). Increase was no longer observed if transporter density was set to 10000/µm^2^.

### Diffusion barriers versus uptake: effects on glutamate spill-out

**Figure 4:**
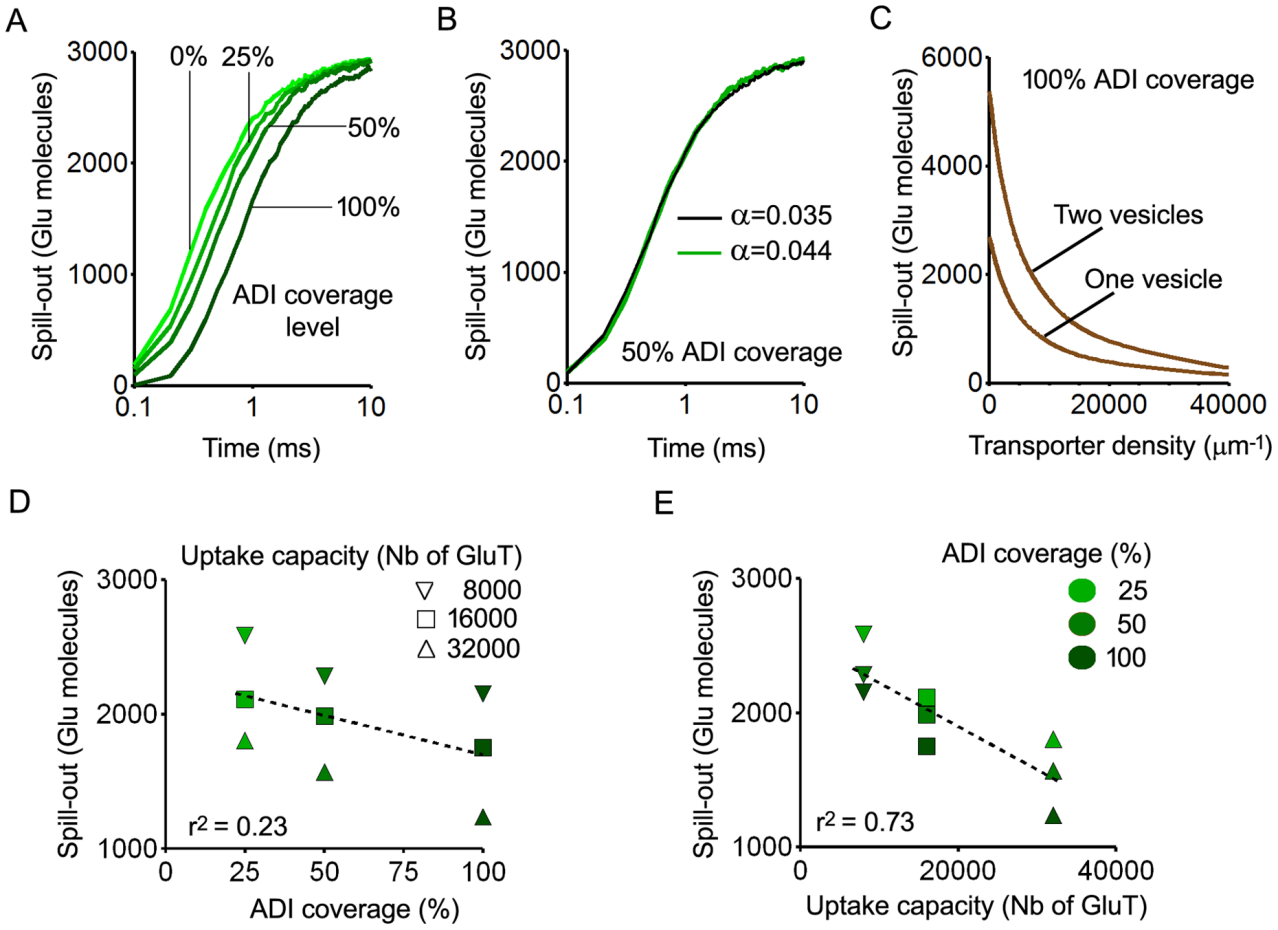
Effects of diffusion barriers and uptake capacity on glutamate spill-out. A. Time course of glutamate exit from the synapse and its immediate vicinity in different conditions of ADI coverage without uptake. Increasing the proportion of ADI perimeter covered by glia delays spill-out. B. Time course of glutamate exit from the synapse and its immediate vicinity in conditions of 50% ADI coverage without uptake. The two different arrangements of glial barriers depicted in Figure 2B and C resulting in different porosity values (α = 0.044 and α = 0.035) were tested and produced nearly identical exit rates. C. Effects of uptake in conditions of full ADI coverage: final amounts of glutamate escaping the synapse as a function of transporter density. The ratio between the curves obtained for the release of one vesicle and two vesicles is a constant (0.5) indicating that no saturation occurs even with the lowest transporter densities. D and E. Regression analysis showing the respective roles of diffusion barriers and uptake in preventing spill-out.

Diffusion barriers and uptake have opposite effects on cleft glutamate content but cooperate in preventing glutamate spill-out, i.e. the long range diffusion of glutamate molecules away from synapses. To determine how these two factors interact in limiting long range glutamate diffusion, we measured spill-out levels using various extents of glial wrapping and transporter densities. Spill-out was defined as the number of glutamate molecules laying outside the synapse and its immediate vicinity (i.e. outside the ADI and the portion of the hollow cylinder within 1000 nm from the ADI border on each side). Glial wrapping was similar to that depicted in Figure 3A but surrounded different portions of the ADI perimeter: the full circumference (100% coverage), one half of it (50% coverage) or one quarter of it (25% coverage). For each level of coverage, trials were performed with different uptake capacities obtained by adjusting the density of transporters in glial membranes (between 1000/µm^2^ and 40000/µm^2^). As expected, increasing ADI coverage without allowing any uptake to occur delayed glutamate exit from the synapse but did not change the final amount of spill-out (Figure 4A). For a given level of ADI coverage, glutamate exit rates were identical with or without actual reduction of extracellular volume fraction (Figure 4B). The final amount of glutamate that escaped the synapse decreased with increasing uptake capacity (Figure 4C). No saturation occurred. This was shown by the fact that, for a given transporter density, the amount of spill-out was proportional to the number of glutamate molecules released (3000 for one vesicle and 6000 for two vesicles). The decrease in spill-out with increased uptake capacity was asymptotic. Thus, glutamate escape could not be entirely prevented, even by very high transporter densities (>20000/µm^2^). Since delayed glutamate escape due to ADI coverage may enhance uptake, regression analysis was performed to determine how the two parameters interacted (Figure 4D,E). The final amount of glutamate that escaped the immediate synaptic environment (averaged value between 4 and 5 ms after release) was best predicted by uptake capacity (i.e. the total number of transporter sites present in the model, r^2^ = 0.73) than by the level of ADI coverage (r^2^ = 0.23). This suggests that diffusion barriers have an accessory role in preventing spill-out.

### Receptor responses

There is large body of evidence indicating that glutamate receptors are present both within and outside synapses. However, analysis was restricted here to postsynaptic receptors located within the ADI (synaptic and non-synaptic parts). The effects of glia were evaluated by calculating AMPAR and NMDAR *P_open_* and mGluR1 activation ratio under three different conditions: (i) without diffusion barriers and uptake, (ii) with diffusion barriers as in Figure 3A (full ADI coverage) but no uptake and (iii) with both diffusion barriers and transporters (10000/µm^2^).

**Figure 5:**
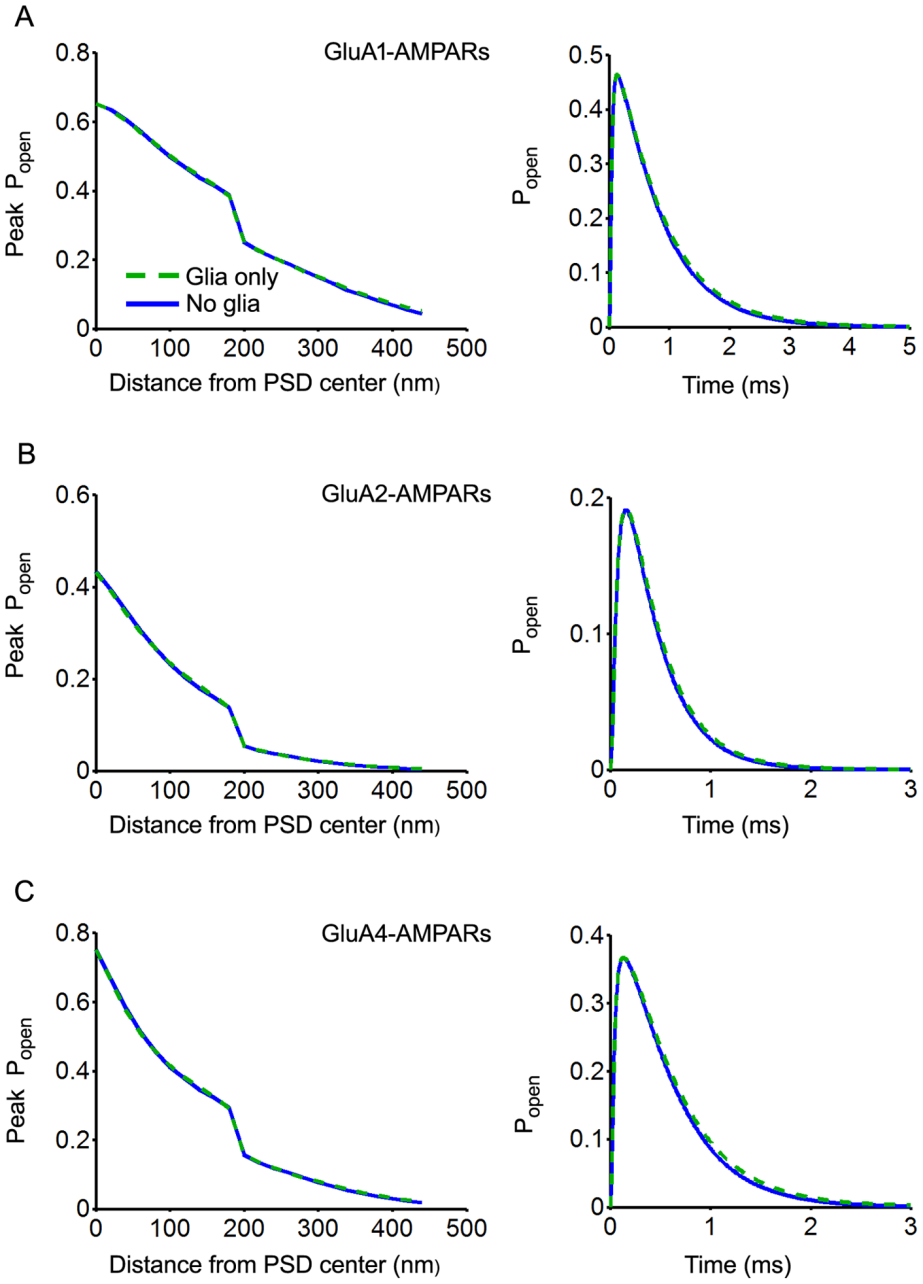
Effects of glial wrapping on AMPAR activation (model as in Figure 3A, full ADI coverage).

AMPAR are homo- or hetero-tetramers and bear 4 binding sites (one per subunit). AMPAR subunits are termed GluA1 to GluA4. GluA3 was not included in the present study since there was no published kinetic scheme and rate constants available for this subunit. The effect of receptor location was first investigated. Whatever the subunit – GluA1, GluA2 or GluA4– the amplitude of the response (peak *P_open_*) was found to sharply decrease with distance to PSD center/release site. An abrupt fall was observed at the edge of the PSD reflecting faster glutamate diffusion in the non-synaptic part of the ADI ( D set to 0.75 µm^2^.ms^−1^ versus 0.4 µm^2^.ms^−1^ in the cleft). Whatever the subunit and its location, the amplitude of the response was unchanged after incorporation of diffusion barriers (Figure 5A–C ; left and right panels) and transporters (not shown). This result was expected not only because AMPAR have a low affinity that prevent detection of small changes in glutamate concentrations but also because of their fast kinetic. Indeed, depending on the subunit and its location, peak *P_open_* was reached within 0.1–0.4 ms after release, i.e. before any change in cleft glutamate content occurred in the presence of glia and/or transporters (see Figure 3B,C). Possible effects of glia on the decay of the responses were assessed by calculating the average *P_open_* of receptors located in the PSD (synaptic receptors) assuming a homogeneous distribution. Adding diffusion barriers in the model had almost no effect on the kinetics of GluA1-, GluA2- and GluA4-AMPAR responses (Figure 5A–C: right panels).

**Figure 6:**
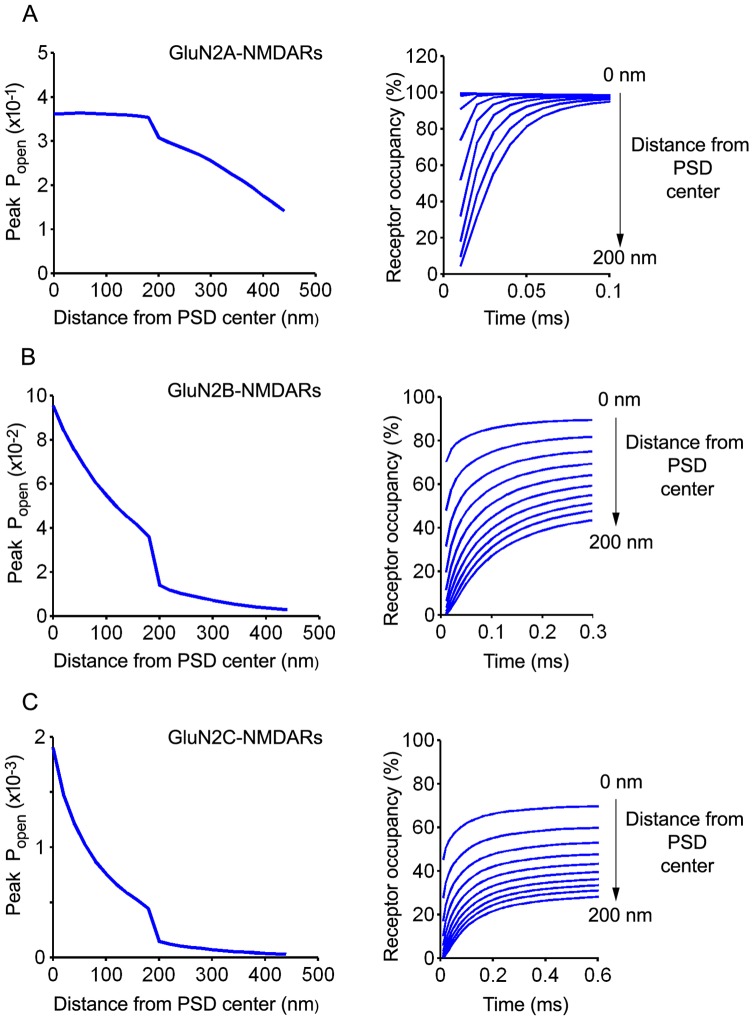
NMDAR responses in control conditions (no glia). A, B, and C are results obtained using GluN2A, GluN2B and GluN2C kinetic schemes, respectively. Left panels represent receptor P_open_ (peak values) as a function of distance to release site. Peak activations of GluN2B and GluN2C receptors sharply decrease with distance to release site whereas GluN2A receptors have a nearly identical peak P_open_ throughout the PSD. Right panels describe receptor occupancy time course at various distances from release site. The 10 successive blue curves in each panel were obtained by 20 nm increments in receptor location from PSD center to periphery. Note that GluN2A receptors undergo rapid nearly complete saturation whatever their location in the PSD.

NMDAR are hetero-tetramers made of 2 GluN1 subunits that bear glycine binding sites and 2 GluN2 subunits that bear glutamate binding sites. There are 4 GluN2 subunits termed GluN2A to D. GluN2D was not included in the present study since there was no published kinetic scheme and rate constants available for this subunit. In agreement with previous observations made by Santucci and Raghavachari [21], the response of GluN2A-NMDAR was found to be less dependent on location with respect to release site than those of GluN2B- or GluN2C-NMDAR (Figure 6A–C, left panels). Whereas peak *P_open_* of GluN2B- or GluN2C-NMDAR sharply decreased with distance from PSD center, peak *P_open_* of GluN2A-NMDAR was nearly constant throughout the PSD and decreased progressively beyond PSD edges. Analyzing the time-course of receptor occupancy showed that GluN2A-NMDAR located in the PSD became near saturated (95–99% occupancy) within 10–100 µs after release depending on distance to PSD center (Figure 6A, right panel). On the contrary, the occupancies of GluN2B- and GluN2C-NMDAR located in the PSD increased slowly after release (Figure 6B,C ; right panels) and became maximal between 1 and 8 ms depending on receptor type and location (not shown). Peak levels of occupancy ranged between 50% and 90% for GluN2B-NMDAR and between 30 and 70% for GluN2C-NMDAR depending on receptor location. Thus, contrary to synaptic GluN2A-NMDAR, synaptic GluN2B- and GluN2C-NMDAR were far from saturation after single vesicle release in spite of their higher steady-state affinities toward glutamate (Kd: 6 and 13 µM, respectively, versus 32 µM for GluN2A-NMDAR).

**Figure 7:**
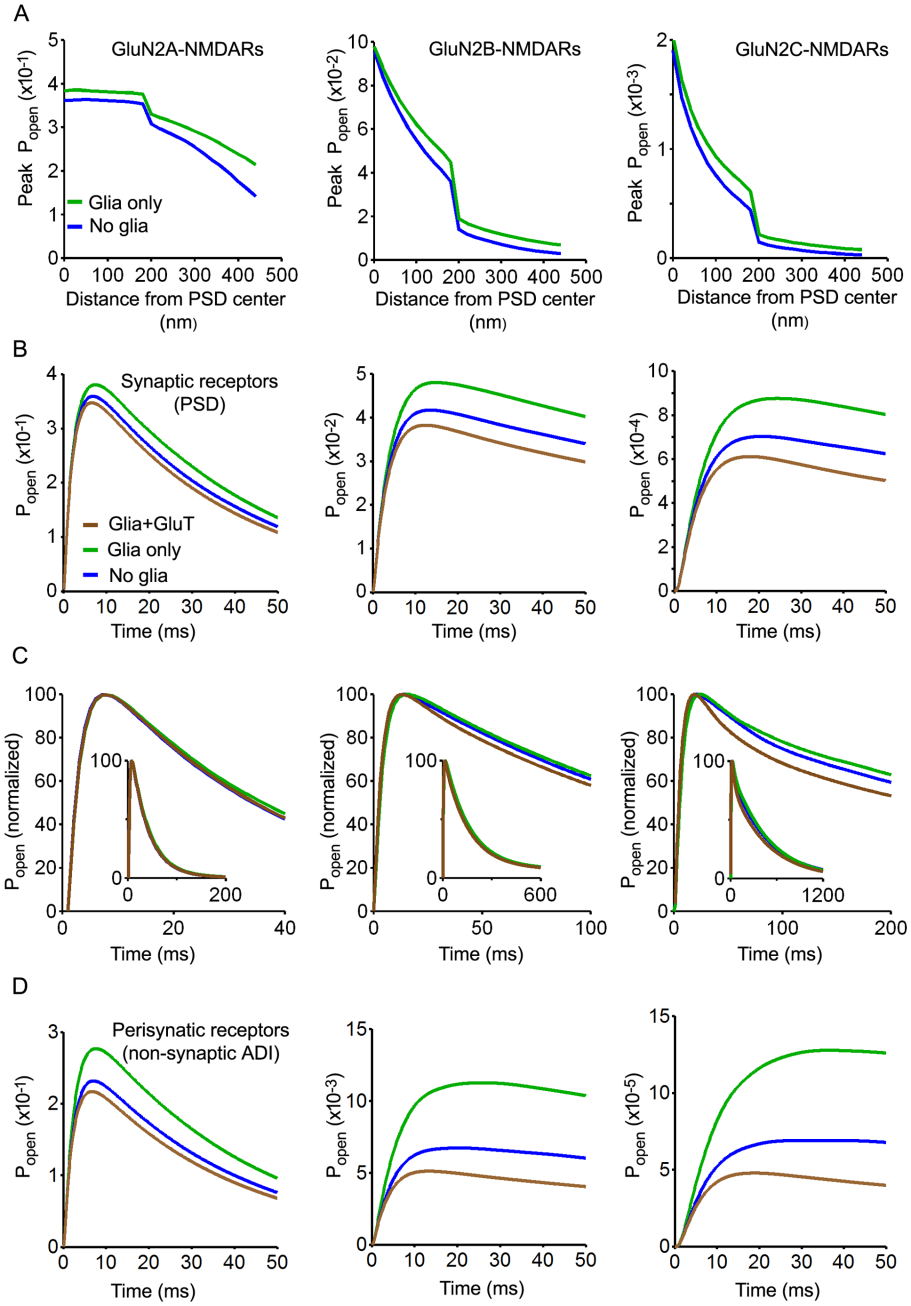
Effects of glial wrapping on NMDAR responses (model as in Figure 3A, full ADI coverage). Left, middle and rigth panels in each row correspond to results obtained with GluN2A, GluN2B and GluN2C kinetic schemes respectively. A. Receptor P_open_ (peak value) as a function of distance to release site in control conditions (no glia, blue) and with diffusion barriers but without uptake (glia only, green). B. Changes in synaptic receptor response (averaged P_open_ assuming an homogeneous receptor distribution in the PSD) induced by the presence of diffusion barriers (green) and by diffusion barrier with transporters (brown ; 10000/µm^2^). GluN2A receptor are only marginally affected whereas GluN2B and GluN2C receptor responses exhibit substantial increases or decreases. C. Normalization of data shown in B illustrating changes in the kinetic of the response induced by diffusion barriers (green) and by diffusion barrier with transporters (brown ; 10000/µm^2^). D. Changes in perisynaptic receptor responses (averaged P_open_ assuming an homogeneous receptor distribution in the non-synaptic part of the ADI) induced by the presence of diffusion barriers (green) and by diffusion barrier with transporters (brown ; 10000/µm^2^).

Contrary to AMPAR, all 3 NMDAR subtypes responded to changes in glutamate concentrations induced by the presence of glia and transporters. Addition of diffusion barriers increased the peak *P_open_* of GluN2A-, GluN2B- and GluN2C-NMDAR (Figure 7A). Increase in peak *P_open_* occurred whatever the receptor location within or outside the PSD but its relative magnitude increased with distance to release site. Standard synaptic responses were obtained for each receptor subtype by averaging the *P_open_* obtained across the PSD assuming a homogenous distribution in the PSD (Figure 7B). Increase in peak *P_open_* induced by diffusion barriers was stronger for synaptic GluN2B- and GluN2C-NMDAR (15% and 25%, respectively) than for synaptic GluN2A-NMDAR (6%). The fact that GluN2B- and GluN2C-NMDAR were far from saturation after single vesicle release, contrary to GluN2A-NMDAR, explains their higher sensitivity to changes in the time course of glutamate in the cleft. Importantly, subsequent insertion of transporters (10000/µm^2^) showed that, whatever the receptor subtype, the increases in peak *P_open_* induced by diffusion barriers could be fully reversed by uptake (Figure 7B). Introduction of diffusion barriers and transporters also modified the kinetic of the response, especially for GluN2B and GluN2C-NMDAR (Figure 7C). Since NMDAR also exist outside synapses, effects of glia on perisynaptic NMDAR were investigated. Standard responses were obtained for each receptor subtype by averaging the *P_open_* obtained across the non-synaptic ADI assuming a homogenous distribution (Figure 7D). Increases in peak *P_open_* induced by diffusion barriers were 19%, 67% and 85% for perisynaptic GluN2Am, GluN2B- and GluN2C-NMDAR, respectively.

**Figure 8:**
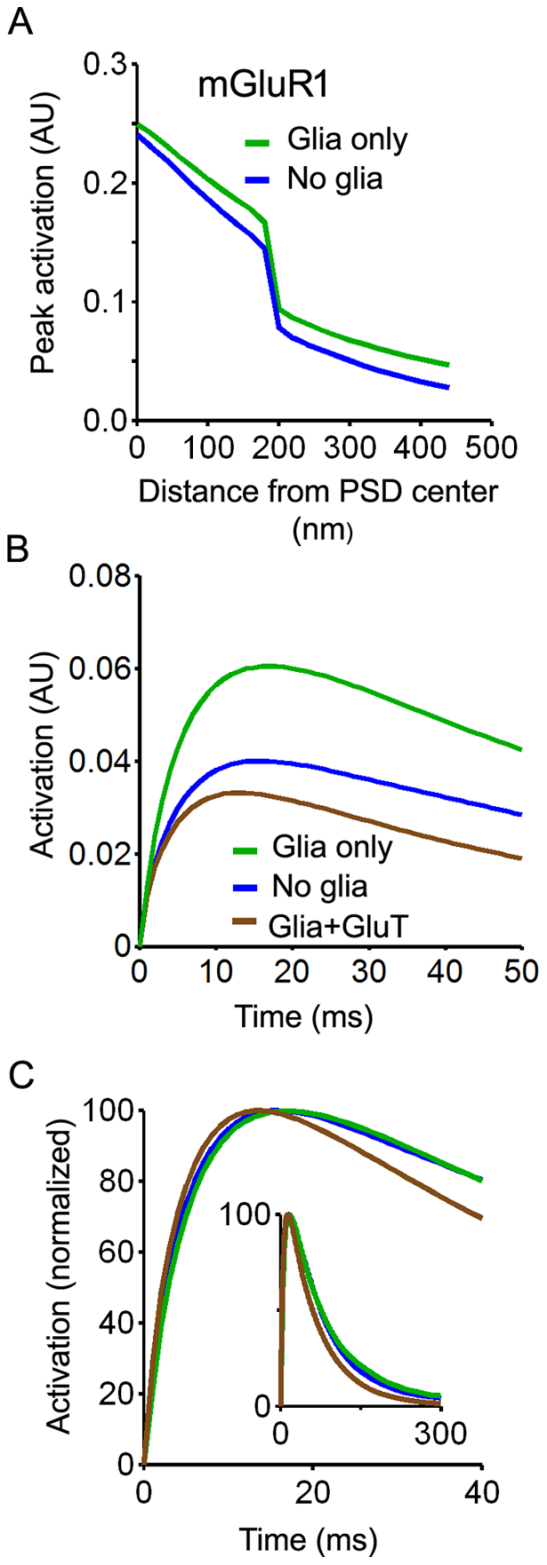
Effects of glial wrapping on mGluR1 receptor responses (model as in Figure 3A, full ADI coverage). A. Receptor activation (peak value, arbitray units) as a function of distance to release site in control conditions (no glia, blue) and with diffusion barriers but without uptake (glia only, green). B. Changes in perisynaptic receptor response (averaged activation assuming an homogeneous receptor distribution in the non-synaptic part of the ADI) induced by the presence of diffusion barriers (green) and by diffusion barrier with transporters (brown ; 10000/µm^2^). C. Changes in response kinetic induced by diffusion barriers without transporters (green) and by diffusion barriers with transporters (brown; 10000/µm^2^).

Results obtained for the mGluR1 receptor, which has a preferential perisynaptic localization, were very similar to those obtained for GluN2B and GluN2C-NMDARs. Introduction of diffusion barriers increased receptor occupancy and activation (+30% for perisynaptic receptors) and this effect was reversed by uptake (Figure 8A,B). Introduction of diffusion barriers also prolonged receptor activation whereas the presence of transporters resulted in accelerated response rise and decay (Figure 8C).

## Discussion

The synapse model used here was designed to reproduce the main characteristics of NTS glutamatergic synapses. Detailed quantitative information provided by electron microscope data was used to construct a complex diffusion space as close as possible to the one existing at actual synapses. This model had some unusual features. First, values used here for cleft width are much smaller than those used in most simulation studies. However, the possibility that the small cleft width measured on electron micrographs resulted from fixation artifacts appears unlikely. Recent data obtained by comparing the effects of tissue processing by aldehyde fixation and high-pressure freezing on the same type of synapses indicate that the shrinkage induced by aldehyde fixation does not affect cleft width [22]. Another unusual feature of the present model is the fact that the cylinder representing the axon-dendrite interface (ADI) was divided into a synaptic and a non-synaptic part having different coefficients for glutamate diffusion. This distinction was based on the previous demonstration that NTS glutamatergic synapses have more than half of their ADI devoid of membrane specialization [4]. They resemble in this respect CA1 hippocampal synapses that also have a large axon-spine apposition that encompasses the PSD area [2]. Electron microscope data indicate that the synaptic cleft is not a free space. The dense material that lies between the pre- and the post-synaptic membranes is not easy to distinguish using conventional electron microscope staining but it is conspicuously visible on EPTA-treated tissue (see for instance [23]). It has also been observed after high-pressure freezing [24]. Furthermore, measurements performed on cryo-electron microscope images of vitrous sections indicate that the concentration of material in the synaptic cleft is higher than in the cytoplasm [25]. No such material seems to exist in the non-synaptic extracellular space since its density is lower than that measured in the cleft [25]. Furthermore, experiments based on the use of specific glycoprotein staining suggest that the extracellular matrix components are more concentrated in the cleft that in the non-synaptic extracellular space [26]. It was thus considered here that diffusion retardation by macromolecular obstacles in the non-synaptic extracellular space was negligible as compared to that occurring in the cleft. On the other hand, no correction was necessary to account for the geometrical (micron-scale) component of tortuosity since the extracellular space was not treated as a porous medium but explicitly represented (see [8] for discussion of this point). For these reasons, the extrasynaptic coefficient of diffusion was set to the free medium value.

This study was performed to determine how perisynaptic glia controls cleft glutamate concentrations and glutamate spill-out. Glial processes act both as physical barriers that oppose diffusion and as sinks that remove transmitter molecules from the extracellular space. A model reproducing the main anatomical features of actual synapses, as the one used in the present study, should correctly predict barrier effects since they mostly depend on the exact disposition of glial processes around synapses. On the contrary, accurately predicting the sink effects of glia would require precise knowledge of the amount of glutamate transporters present in perisynaptic glial membranes. There has been few attempts to measure transporter densities in astrocytic membranes. In their study performed by quantitative immunoblotting and estimates of glial surface densities, Lehre and Danbolt report that transporters densities (Glast + GLT1 densities) in cerebellar and hippocampal astrocytes membranes are close to 5000/µm^2^ and 10000/µm^2^, respectively [27]. However, these density values are averages that also include non-perisynaptic glial membranes. They may thus be lower than actual perisynaptic membrane values. In addition, similarly to what occurs for wrapping levels, transporter concentrations in perisynaptic glia may greatly differ from synapse to synapse in the same brain region. For these reasons, a large range of transporter densities was tested in the present study. Results showed that efficient prevention of spill-out required high number of uptake sites in the vicinity of the synapse. The level of glial wrapping is often interpreted as indicating the possibilities of glutamate escape from the cleft. Intuitively, spill-out appears more likely if a large part of the synaptic diameter is free from glia. However, this view is questioned by the present data since they suggest that spill-out prevention depends more on the total number of transporters being present than on their positioning around the synapse. Substantial glutamate escape occurred at synapses entirely surrounded by glia but with low transporter densities. Conversely, the sink effects induced by high transporter concentrations efficiently prevented spill-out even if most of the synaptic diameter was free from glia. Thus, it may be said that if extensive glial wrapping helps prevent spill-out, it does so by bringing more transporters close to the synapse rather than by creating diffusion barriers all around the cleft.

Uptake and diffusion barriers act synergistically to prevent spill-out but are expected to have opposite effects on cleft glutamate concentrations. Accordingly, it was shown here that the closure of escape routes by glia delayed glutamate exit from the cleft. However, contrary to the conclusions of Rusakov [5], it was also found that this effect was compensated by uptake resulting from realistic transporter densities. Without uptake, the prolonged presence of glutamate in the cleft had no effect on AMPAR activation but increased the response of NMDAR and metabotropic receptors. Consistent with the present results, several studies indicate that uptake blockade by glutamate transporter antagonists has no effect on AMPAR currents but significantly increases NMDAR currents [7],[8],[28],[29]. The prevailing view is that the effects of transporter antagonists are mostly due to increased glutamate spill-out allowing distant activation of extrasynaptic NMDAR and of NMDAR located in neighbouring synapses. The present simulation data indicate that increased NMDAR currents after transport blockade may also result from enhanced activation of synaptic receptors, provided that these receptors are not saturated in basal conditions. It may be the case if these receptors are enriched with the GluN2B and/or GluN2C subunits which have slow binding kinetics preventing rapid saturation.

Numerous synapses in the CNS express NMDAR that are not saturated by single vesicle release [30],[31],[32]. According to the present results, glutamate transporters expressed in perisynaptic glia could control the activation of these synaptic NMDAR. This mechanism would allow differential tuning of NMDAR subtypes according to their subunit composition since changes in transporter densities would selectively affect the “slow” GluN2B and GluN2C receptors leaving the “fast” GluN2A receptor responses merely unchanged. There is evidence showing that the different GluN2 subunits have different functionality via their differential interactions with MAGUK proteins and downstream signaling pathways [33],[34]. Changes in transporter densities expressed by perisynaptic glial membranes would therefore permit differential activation of these signaling pathways.
